# Convolutional neural network for automated tooth segmentation on intraoral scans

**DOI:** 10.1186/s12903-024-04582-2

**Published:** 2024-07-16

**Authors:** Xiaotong Wang, Khalid Ayidh Alqahtani, Tom Van den Bogaert, Sohaib Shujaat, Reinhilde Jacobs, Eman Shaheen

**Affiliations:** 1 OMFS IMPATH Research Group, Department of Imaging and Pathology, Faculty of Medicine, KU Leuven, Kapucijnenvoer 33, Leuven, 3000 Belgium; 2 Department of Oral and Maxillofacial Surgery, The First Affiliated Hospital of Harbin Medical University, Youzheng Street 23, Nangang, Harbin, 150001 China; 3 Department of Oral and Maxillofacial Surgery and Diagnostic Sciences, College of Dentistry, Sattam Bin Abdulaziz University, Al-Kharj, 16278 Saudi Arabia; 4 King Abdullah International Medical Research Center, Department of Maxillofacial Surgery and Diagnostic Sciences, College of Dentistry, King Saud bin Abdulaziz University for Health Sciences, Ministry of National Guard Health Affairs, Riyadh, 14611 Saudi Arabia; 5 Department of Oral and Maxillofacial Surgery, University Hospitals Leuven, Kapucijnenvoer 33, Leuven, 3000 Belgium; 6 Department of Dental Medicine, Karolinska Institutet, Solnavägen 1, 171 77, stockholm, 3000 Sweden

**Keywords:** Artificial intelligence, Machine learning, Neural networks, Computer, Optical imaging, Dentition, Dentistry

## Abstract

**Background:**

Tooth segmentation on intraoral scanned (IOS) data is a prerequisite for clinical applications in digital workflows. Current state-of-the-art methods lack the robustness to handle variability in dental conditions. This study aims to propose and evaluate the performance of a convolutional neural network (CNN) model for automatic tooth segmentation on IOS images.

**Methods:**

A dataset of 761 IOS images (380 upper jaws, 381 lower jaws) was acquired using an intraoral scanner. The inclusion criteria included a full set of permanent teeth, teeth with orthodontic brackets, and partially edentulous dentition. A multi-step 3D U-Net pipeline was designed for automated tooth segmentation on IOS images. The model’s performance was assessed in terms of time and accuracy. Additionally, the model was deployed on an online cloud-based platform, where a separate subsample of 18 IOS images was used to test the clinical applicability of the model by comparing three modes of segmentation: automated artificial intelligence-driven (A-AI), refined (R-AI), and semi-automatic (SA) segmentation.

**Results:**

The average time for automated segmentation was 31.7 ± 8.1 s per jaw. The CNN model achieved an Intersection over Union (IoU) score of 91%, with the full set of teeth achieving the highest performance and the partially edentulous group scoring the lowest. In terms of clinical applicability, SA took an average of 860.4 s per case, whereas R-AI showed a 2.6-fold decrease in time (328.5 s). Furthermore, R-AI offered higher performance and reliability compared to SA, regardless of the dentition group.

**Conclusions:**

The 3D U-Net pipeline was accurate, efficient, and consistent for automatic tooth segmentation on IOS images. The online cloud-based platform could serve as a viable alternative for IOS segmentation.

## Background

Conventional dental impression techniques have been largely replaced by digital intraoral scanning, which offers greater precision, is non-invasive, and provides increased patient comfort [1]. Integrating intraoral scanned (IOS) data into digital workflows for prosthodontics, orthodontics, implant dentistry, and orthognathic surgery has significantly enhanced treatment planning efficiency and simplified clinical procedures by eliminating labor-intensive and time-consuming steps associated with conventional impressions [2].

A crucial step in digital dental workflows is the three-dimensional (3D) segmentation of teeth from the IOS dataset. Accurate and efficient tooth segmentation is essential for clinical applications that require tooth realignment for treatment simulation or follow-up evaluation, such as in orthodontics and implantology [3–5]. This accuracy is vital for achieving reliable and stable treatment outcomes [6].

Currently, semi-automatic segmentation algorithms in imaging software are still the preferred methods for segmenting teeth on IOS images. These algorithms extract geometric features such as surface contour lines, surface curvature, and harmonic field from the IOS data [7–9]. Despite being widely used in digital dental workflows, semi-automatic segmentation has limitations, including a lack of robustness, the need for manual correction, labor intensiveness, dependence on expertise, and excessive time consumption. To address these issues, significant efforts have been made to develop automatic segmentation tools. However, this remains challenging due to substantial variability in IOS data among different patients, including large-scale morphological and geometric variations of teeth, missing or disarranged teeth, and abnormal dental conditions such as supernumerary teeth. Additionally, tooth rotation and crowding complicate the delineation of each tooth’s margins. This challenge is exacerbated in orthodontic patients with dental braces or indistinguishable gingival boundaries [5].

Recently, artificial intelligence (AI) has gained traction in the field of medicine due to its potential to automate tasks mimicking human intelligence [10]. Deep-learning-based convolutional neural networks (CNNs), a subcategory of AI, have been considered the most suitable method for medical image analysis [11–14]. Several studies have successfully employed CNNs to segment teeth from IOS datasets with satisfactory performance [15]. However, these studies often rely on small sample sizes or fail to investigate the networks’ robustness in handling deviations from natural dentition and variability in dental status, such as missing teeth, crowding, or orthodontic brackets [4, 5, 16–19].

Therefore, the aim of the current study was to propose and validate the performance of a CNN model for automatically segmenting teeth on IOS images, including those with a full set of natural teeth, orthodontic brackets, and partially edentulous dentition.

## Methods

This study complied with the World Medical Association Declaration of Helsinki on medical research. This study received ethical approval from the Ethics Committee Research of University Hospitals Leuven (reference number: S65188) and followed the Artificial Intelligence in Dental Research checklist (Appendix Table 1) [20].

### Dataset

The dataset included 761 IOS images (380 upper jaws and 381 lower jaws) acquired by a Trios 3Shape intraoral scanner (Copenhagen, Denmark) between June 2020 and April 2021 from the LORTHOG Register, Department of Oral & Maxillofacial Surgery, University Hospitals Leuven. All the data were retrospectively collected and anonymized. The inclusion criteria were complete scans of jaws with a full set of permanent teeth, orthodontic patients with brackets, and prosthodontic patients with partially edentulous dentition. Any local pathological conditions were excluded. The dataset was randomly divided into three subsets for training (*n* = 609), validation (*n* = 76), and testing (*n* = 76).

### AI model architecture

**Fig. 1:**
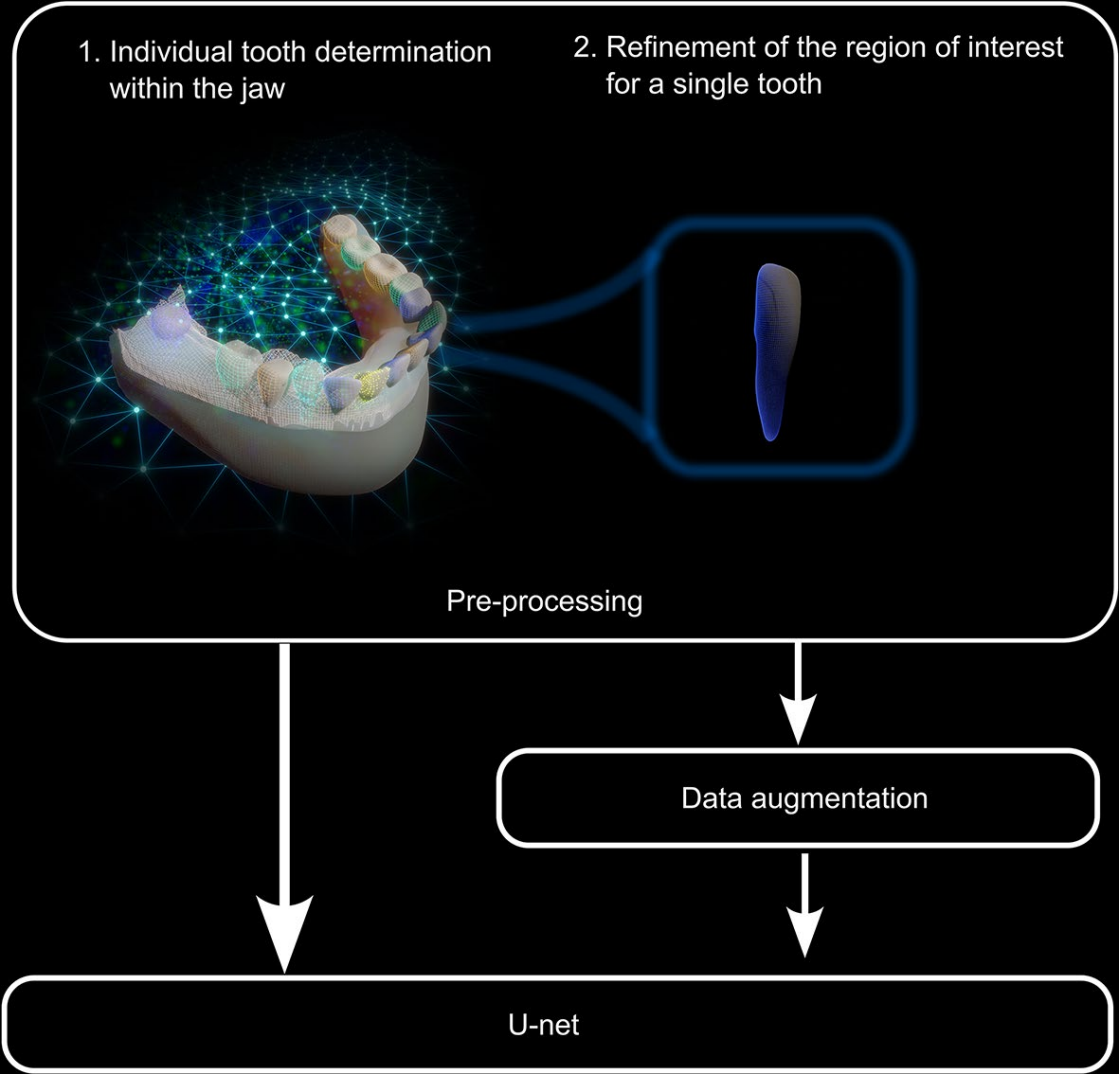
The multi-step approach using U-Net models

A multi-step 3D U-Net pipeline was designed for automated tooth segmentation on IOS images. While a basic 3D U-Net network was implemented [21], minor adjustments to the hyperparameters were made to prevent overfitting and optimize evaluation metrics such as the IoU, Dice coefficient score, and 95% HD. The proposed multi-step approach using U-Net models aims to refine tooth segmentation by improving the data quality and increasing the training dataset size through data augmentation (Fig. 1), resulting in more accurate and robust segmentation. Below is a detailed explanation of each stage:

#### Preprocessing the Raw STL data

##### Region of interest (ROI) extraction

 The ROI of the tooth structure was extracted from the raw STL data. The ground truth datasets were labeled by human experts. IOS data were prepared by semi-automatic segmentation (SA) in OrthoAnalyzer software (3Shape A/S, Copenhagen, Denmark) and exported in standard tessellation language (STL) format. Segmentation tasks were randomly performed by three dental practitioners following initial training and calibration.

The IOS image was first preprocessed by preparing a model set and then assigned to the segmentation field. Missing teeth were deselected, followed by manual indication of distal and mesial points to create a cut spline outlining the tooth contour. Then, a sculpt was created, and the toolkit for addition or removal was used for minor correction of over- or under-estimations in the segmentations. Extra corrections were applied for cases with brackets by removing the connecting wire and isolating the teeth. Finally, the segmented teeth were labeled according to FDI notation using 3-matic 14.0 software (Materialise, Leuven, Belgium). All segmentations and labels were checked by a second observer for quality control, with necessary alterations made as needed.

##### Smoothing

To improve the quality of the ROI, monochromatic data were smoothed using techniques such as smoothing filters, morphological operations, and level set methods. These operations primarily focused on refining image texture and reducing noise. Specifically, Gaussian blurring and median filtering were employed. Gaussian blurring involved convolving the image with a Gaussian kernel to achieve a smoother appearance, effectively reducing high-frequency noise. Median filtering was used to reduce impulse noise by replacing pixel values with the median value of neighboring pixels. These smoothing operations were selected to enhance image quality and prepare it for subsequent analysis and processing stages.

#### Data augmentation

To increase the size and variability of the training dataset, data augmentation techniques were applied to the preprocessed ROI data. This included operations such as scaling, rotation, flipping, and deformations, which created new training samples from the original data. Data augmentation improves the model’s generalization performance by making it more robust to variations and artifacts in the input data. The Adam optimizer, an adaptive learning rate optimization algorithm, was used to train the U-Net networks.

To apply U-Net to mesh data, several preprocessing steps were followed to create a suitable input for the neural network:

#### Voxelization

The STL mesh file was converted into a volumetric representation through voxelization. The process of voxelization for the STL files involved converting the continuous surface geometry into a discrete volumetric representation. This was achieved by partitioning the STL mesh into a three-dimensional grid of equally sized cubic elements, known as voxels. Each voxel was assigned a value based on its intersection with the mesh surface. The voxelization procedure included the following steps: grid generation, intersection testing, voxel value assignment, and volumetric data creation.

#### Patch division

The volumetric representation was divided into smaller sub-volumes, called patches. Each patch was used as input to the network.

#### Feature extraction

Features were extracted for each patch using convolutional layers. In U-Net, the encoder part consists of a series of convolutional layers with pooling operations to extract high-level features from the input.

#### Symmetric Decoder

A symmetric decoder architecture was used to reconstruct the output segmentation. The U-Net decoder includes a series of up convolutional layers with skip connections from the encoder part to reconstruct the segmentation.

#### Training

Binary cross-entropy was used as the loss function to train the U-Net on labeled mesh data. The labels for each patch were obtained by applying a labeling process to the mesh.

Once trained, the U-Net can predict the segmentation for new mesh data patches.

### Validation metrics

The performance of the CNN model was evaluated using the following metrics:

- Intersection over Union (IoU):

$$\:IoU=\frac{TP}{(TP+FP+FN)}$$

- Timing: The AI runtime for each segmentation was recorded in seconds.
- Dice similarity coefficient (DSC):

$$\:\:DSC=\frac{2\times\:TP}{\left(TP+FP\right)+(TP+FN)}$$

TP = true positives; TN = true negatives; FP = false positives; FN = false negatives.

When applied to mesh data, IoU and DSC metrics were used to compare the overlap between two sets of triangles or polygons. To calculate these metrics, the labeled area of interest was compared to the ground truth or reference data. The mesh data were represented as a collection of vertices and faces. To apply IoU or DSC, the labeled regions on the mesh were converted into a binary mask, where each vertex was either labeled “inside” or “outside” of the labeled region. Specifically, this was done by projecting the 3D model onto a 2D plane and creating a binary image mask using traditional image segmentation methods. For crown surface datasets, typically only the visible or labeled side of the crown was analyzed, with the unlabeled side being ignored during the calculation of the IoU and DSC since it was not part of the labeled area of interest. When calculating these metrics, only the labeled regions were taken into account. Unlabeled regions were ignored, and their contribution to the final metric score was treated as if they were correctly segmented. It is important to note that the accuracy of the evaluation metrics can be affected by the quality of the labeling and segmentation process, as well as the specific characteristics of the mesh data being analyzed.

### Clinical applicability of the CNN model

The CNN model was implemented on an online cloud-based platform (Virtual Patient Creator, Relu Inc, Leuven, Belgium), allowing users to upload STL files of IOS data for automated AI-driven segmentation (A-AI). The platform also offers users tools for correction and generating refined AI-driven segmentation (R-AI). To evaluate the clinical applicability of the tool, an additional subsample of 18 IOS images was tested, including cases with a full set of permanent teeth (*n* = 6), teeth with orthodontic brackets (*n* = 6), and partially edentulous dentition (*n* = 6). The timing, consistency, and accuracy of the A-AI and R-AI segmentations were compared to the semi-automatic (SA) method.

The time for the SA method was measured from importing the STL data into OrthoAnalyzer until the generation of a segmented model. For A-AI, the algorithm automatically calculated the time, while the R-AI time was the sum of the A-AI and the subsequent refinements. Two independent observers performed the SA and R-AI segmentations to assess the inter-observer reliability. To evaluate intra-observer variability, one observer repeated the same segmentations after an interval of two weeks. Furthermore, the accuracies of A-AI and R-AI were compared with SA-based segmentation. The hardware specifications are listed in Table 1.

**Table 1:** Specifications of hardware devices

• Model name: AMD Ryzen 7 3700X	• Model name: NVIDIA GeForce RTX 3060
• Number of CPU cores: 8	• CUDA cores: 3584
• Number of threads: 16	• Total memory: 12GB
• Base clock: 3.6 GHz	
• L1/L2/L3 cache: 512KB/4MB/32MB	
• Total memory: 32GB	

CPU: Central processing unit, GPU: Graphics processing unit, CUDA: Compute unified device architecture

### Statistical analyses

Data were analyzed using IBM SPSS Statistics for Windows, version 21.0 (IBM Corp., Armonk, NY, USA). Descriptive statistics were calculated for each evaluation metric. Normality was assessed using normal quantile plots, and log-transformation was applied to normally distributed data. The intra-class correlation coefficient (ICC) was used to calculate the inter- and intra-observer reliability. Test-retest reliability was also determined. Timing was compared between different methods using two-way repeated measure ANOVA [22]. A p-value of < 0.05 was considered statistically significant.

## Results

### AI model performance

**Fig. 2:**
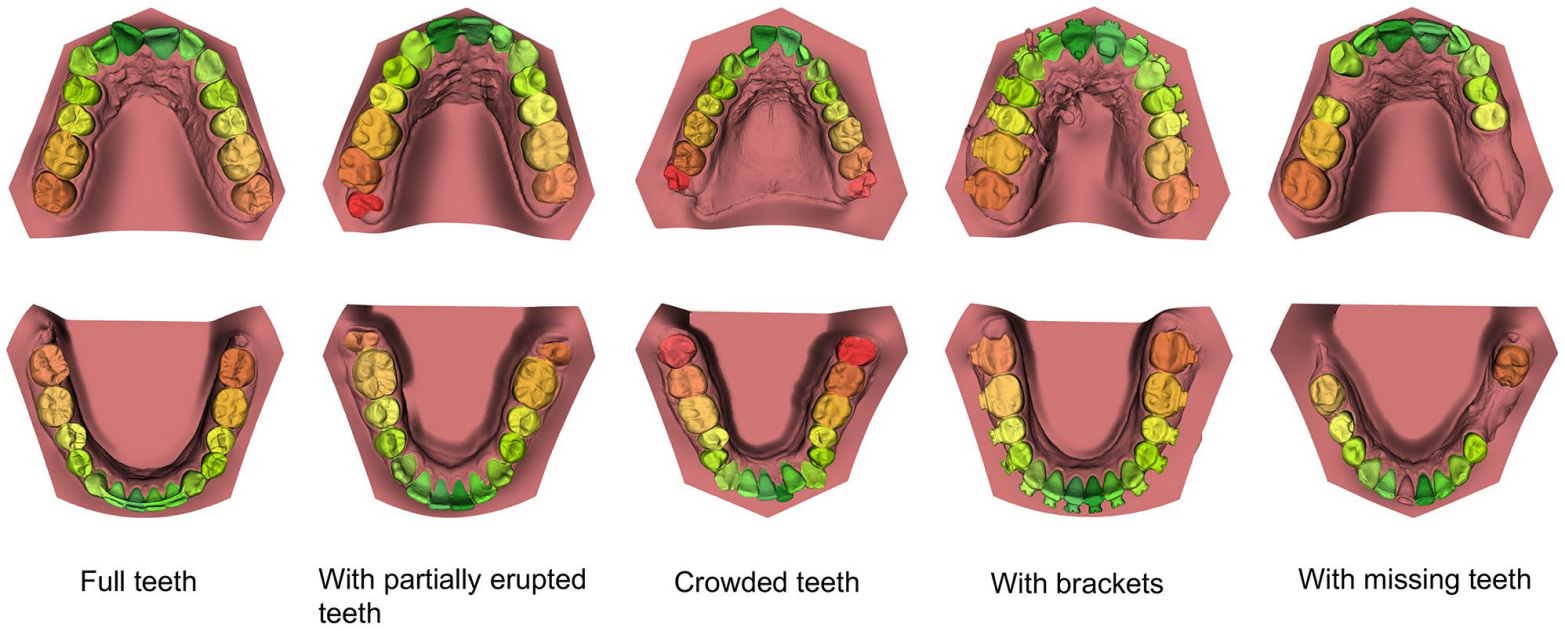
Example of AI segmentation results of the upper and lower jaws for the different dentition groups

Within the 76 validation scans, three groups were identified: full set of teeth (*n* = 41), teeth with brackets (*n* = 13), and partially edentulous dentition (*n* = 22). Table 2 provides an overall view of the CNN model’s segmentation performance compared to the ground truth and the results for each individual group. The CNN model required 31.7 ± 8.1 s per jaw for segmentation, regardless of the dentition group. The model achieved an IoU of 91.0 ± 5.5% and a DSC of 94.6 ± 4.8%, indicating an optimal overlap compared to the ground truth. Among the individual dentition groups, the full set of teeth achieved the highest performance metrics, while the partially edentulous group scored the lowest. Figure 2 illustrates examples of automated segmentations for different dentition types. The CNN model effectively generated dentition with lingual fixed retainers, brackets, and partially erupted, crowded, or missing teeth. Although the segmentation of crowded teeth was optimal, further improvements are needed to enhance the CNN model’s ability to distinguish boundaries in extreme crowding cases.

**Table 2:** The segmentation performance of the AI model (mean ± SD) compared to ground-truth

Dentition group	IoU (%)	DSC (%)	Timing* (s)
Full teeth	92.2 ± 3.8	95.5 ± 3.2	33.0 ± 7.4
Partially edentulous	89.3 ± 8.0	93.0 ± 7.5	31.2 ± 10.6
Brackets	90.0 ± 3.4	94.6 ± 2.0	28.8 ± 2.5
Average	91.0 ± 5.5	94.6 ± 4.8	31.7 ± 8.1

*Note* * Timing for AI segmentation per upper jaw or lower jaw

DSC, Dice coefficient score; IoU, Intersection over union; SD, standard deviation

### Clinical applicability

The average segmentation timing is presented in Table 3. The A-AI method required 66.7 s per case (both upper and lower jaw), while the SA approach took an average of 860.4 s (14.3 min). The R-AI segmentation took 328.5 s, representing a 2.6-fold decrease compared to the SA approach. Two-way repeated measures ANOVA showed a significant interaction between the applied method and operator (*p = 0.02*). A significant difference in timing was observed between methods (SA vs. R-AI) for both operators (*p < 0.001*). The R-AI timing was significantly different between the observers (*p = 0.04*), whereas no significant difference was found with the SA approach (*p = 0.13*).

**Table 3:** Timing of segmentation methods

Method	Mean (s)	SD (s)	Min (s)	Max (s)
SA	860.4	211.4	551.0	1348.0
A-AI	66.7	8.5	55.3	79.5
R-AI	328.3	181.1	101.6	739.5

*Abbreviations* A-AI, automated artificial intelligence-driven segmentation; R-AI, refined artificial intelligence-driven segmentation; SA, semi-automatic method; Max, maximal value; Min, minimal value; SD, standard deviation; s, seconds

The test-retest reliability indicated a high correlation (*r* = 0.873) [23]. Both the intra- and inter-operator reliability of the SA and R-AI were excellent, suggesting a high consistency of the training dataset. As shown in Table 4, the R-AI exhibited higher observer reliability compared to the SA, irrespective of the dentition group, further validating the tool’s effectiveness in performing reproducible and superior segmentation compared to the conventional SA approach.

**Table 4:** Inter and intra-observer assessment based on the ICCs in terms of the IoU (%) for the SA and R-AI methods

Intra-operator consistency		
Dentition group	SA	*R*-AI
Full teeth	93.7	98.2
Partially edentulous	95.4	95.5
Brackets	90.9	98.9
**Inter-operator consistency**		
**Dentition group**	**SA**	**R-AI**
Full teeth	92.9	98.3
Partially edentulous	94.2	97.1
Brackets	91.9	98.2

*Abbreviations* A-AI, automated artificial intelligence-driven segmentation; R-AI, refined artificial intelligence-driven segmentation; SA, semi-automatic method; ICC, Intra-class correlation coefficient; IoU, Intersection over union

**Fig. 3:**
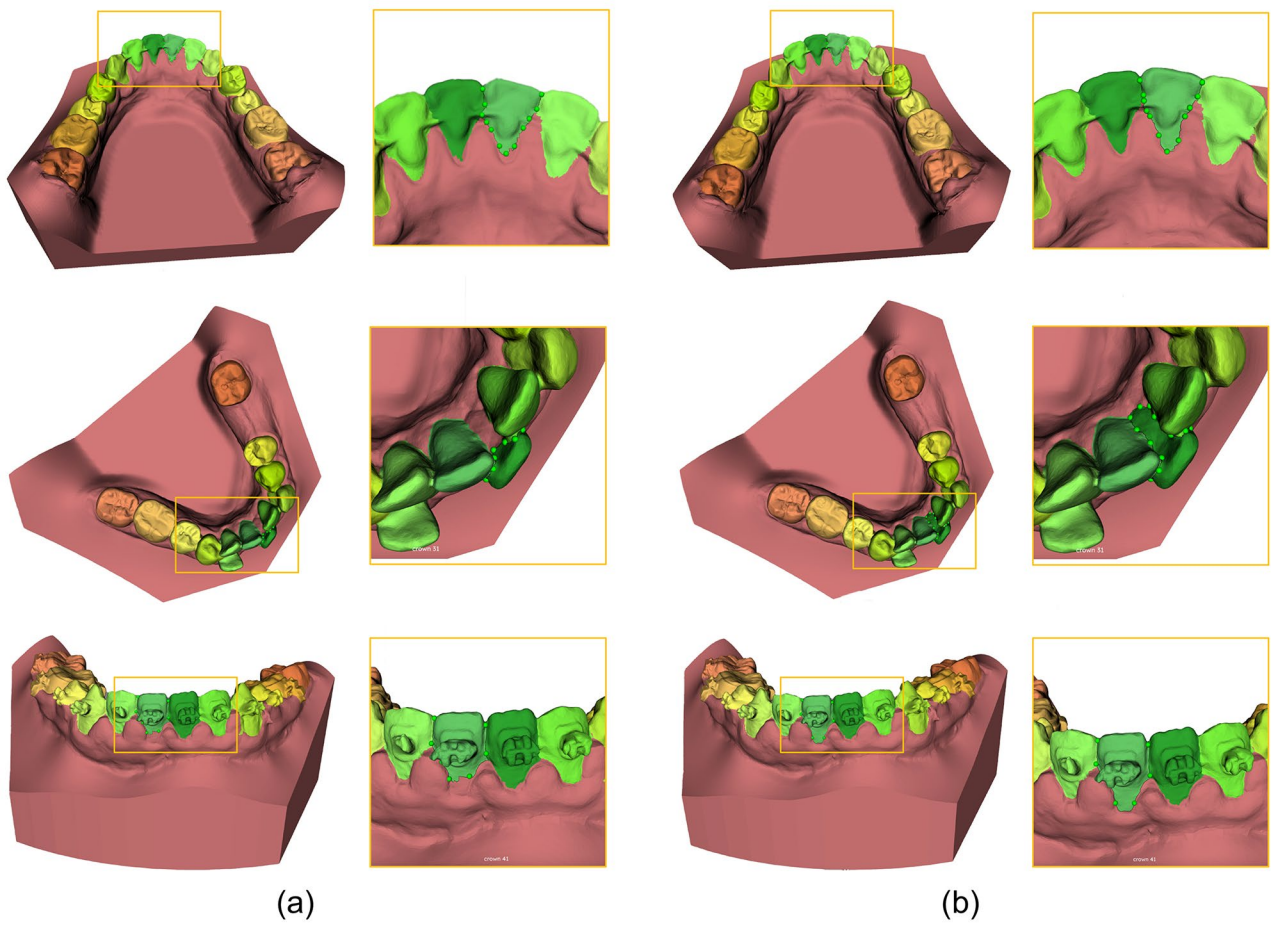
Visual comparison of tooth segmentation with (**a**) automated AI-driven segmentation (A-AI) and (**b**) refined AI-driven segmentation (R-AI). R-AI allowed refinement of the accurate tooth segmentation boundary, as highlighted in the contours

An overview of the accuracy assessment of the A-AI and R-AI compared to the SA for all subgroups is displayed in Table 5. Both A-AI and R-AI achieved high IoUs of 90.5% and 92.5%, respectively. The study demonstrated that R-AI outperformed A-AI in terms of the 95th percentile of the Hausdorff distance (HD), representing the maximum distance between the predicted model and ground truth. The results showed that the bracket group had the highest 95% HD, followed by the full teeth group and the partially edentulous group. A visual illustration of AI segmentation with and without manual refinement is presented in Fig. 3. As shown in the figures, the online platform enabled users to define the boundaries that the AI failed to capture, including areas under lingual fixed retainers, indistinct boundaries around braces and swollen gingiva, and extremely crowded teeth. When incomplete tooth segmentation was observed, manual delineation of the tooth contours was performed. This involved manually adding or removing parts of the tooth mask using the 3D paint brush or 3D contour tool on the AI platform. The operator verified the lingual/palatal, facial/labial, and occlusal surfaces to ensure that the region of interest was accurately masked without any overestimation or underestimation of the margins.

**Table 5:** Accuracy assessment of the A-AI and R-AI vs. the SA methods (mean ± SD)

Metric	Dentition	A-AI vs. SA	*R*-AI vs. SA
IoU (%)	Full teeth	91.3 ± 1.0	94.4 ± 0.8
	Partially edentulous	91.3 ± 3.5	91.8 ± 6.2
	Brackets	88.7 ± 5.4	91.1 ± 6.4
	Average	90.5 ± 4.0	92.5 ± 5.4
DSC (%)	Full teeth	95.4 ± 0.5	97.1 ± 0.4
	Partially edentulous	95.4 ± 2.0	95.6 ± 3.6
	Brackets	93.9 ± 3.1	95.2 ± 3.6
	Average	94.9 ± 2.2	96.0 ± 3.1
95% HD (mm)	Full teeth	0.0030 ± 0.0032	0.0029 ± 0.0033
	Partially edentulous	0.0001 ± 0.0001	0.00006 ± 0.0001
	Brackets	0.7619 ± 1.1795	0.7609 ± 1.1778
	Average	0.2549 ± 0.7384	0.2546 ± 0.7373

*Abbreviations* A-AI, automated artificial intelligence-driven segmentation; R-AI, refined artificial intelligence-driven segmentation; SA, semi-automatic method; DSC, Dice coefficient score; IoU, Intersection over union; SD, standard deviation

## Discussion

In digital dentistry, detecting teeth on IOS images is crucial for treatment planning and follow-up evaluation. In light of recent technological advancements, this study developed a robust fully automated CNN model and provided an easy-to-use interactive tool for IOS tooth segmentation using the Virtual Patient Creator platform. This model ensures time-efficient segmentation with accuracy and consistency. Additionally, the performance of the CNN model was validated for clinical applications by deploying it on an online cloud-based platform where manual corrections could be performed, further facilitating its integration into clinical practice.

Previous studies have not explored the robustness of their proposed automated IOS segmentation algorithms for segmenting teeth with brackets or partially edentulous dentition [5, 16–19]. Therefore, this study included a variety of cases, both normal (full set of teeth) and abnormal (partially edentulous and bracket group), to assess the generalizability of the AI model across a dataset with heterogeneous geometry. The AI model demonstrated high performance in segmenting normal dentition and confirmed its accuracy in complex cases, such as crowded or misaligned teeth, which are typically challenging to identify due to overlapping regions with adjacent teeth [19]. Unlike previous studies, the presented model accurately segmented teeth with brackets and partially edentulous jaws [18]. Furthermore, it outperformed other competing methods in recognizing the boundary between noisy gingiva and partially erupted teeth [24].

To date, various deep learning models have been proposed for tooth segmentation on IOS images, each with different levels of performance. These models have focused mainly on the segmentation of a full set of normal teeth. Zanjani et al. introduced a Mask-MCNet framework, achieving an IoU value of 98%, although this result may be due to their small dataset of 120 IOS images and the unspecified case variability [16]. Lian et al. evaluated MeshSegNet and achieved a DSC value of 95.2% using a dataset of 30 upper-jaw cases, but the model struggled with missing teeth and brackets [18]. Wu et al. proposed the TS-MDL model, which achieved a DSC of 95.3% with a relatively small sample of 36 upper IOS images, although its performance decreased in malocclusion cases [19]. Zhang et al.’s TSGCNet achieved a low IoU of 89% for segmenting incisors [25]. Compared to these studies, our model showed high performance, with a DSC score comparable to or exceeding those of other proposed models [5, 16–19, 23]. One study reported the superior performance of a CNN model (TSegNet) with a DSC of 98% for both normal and abnormal cases. However, the authors did not specify separate DSC scores for these case types or identify the number of abnormal cases. Additionally, their model produced incomplete segmentation of wisdom and rudimentary teeth [24]. In contrast, the 3D U-Net pipeline presented in this study achieved an average DSC of 94.6% across various case groups, confirming its ability to handle both regular and complicated dental morphologies. Importantly, the integration of the CNN model into an online cloud-based platform allows for user refinements (the R-AI method) with interactive tools, enhancing its ability to segment complex dental malformations and confirming its suitability for clinical applications.

Although the presented model demonstrated high performance in automated tooth segmentation, certain error types remain, particularly in low-quality scans and extremely crowded teeth. One reason could be that these cases are quite rare, leading to insufficient learning opportunities for the model. The differences in the 95% HD results between the dentation groups may be due to the complexity and characteristics of different dental conditions. For instance, segmentation errors tend to be more common in cases involving severe malocclusion, indistinct boundaries around the braces, and swollen gingiva. These complexities, which are predominantly found in the brackets group, result in higher deviations from the ground truth. The intricate dental arrangements and overlapping structures present significant challenges for accurate AI segmentation. In contrast, the full teeth group, comprising cases with minor alignment issues or no malocclusion, exhibited fewer deviations. The simplicity and clarity of these cases make them easier for AI to segment accurately. The partially edentulous group, with a few missing teeth, presented the lowest complexity, resulting in the lowest deviations from the ground truth. The straightforward nature of these cases allows for more precise segmentation by the model. The minimal improvement in model performance with R-AI suggests that fewer corrections are needed. R-AI substantially reduces labor-intensive steps, with a 2.6-fold decrease in time consumption and higher consistency compared to the SA method.

Integrating the IOS image segmentation model into the Virtual Patient Creator platform could be a viable tool for planning and follow-up assessments in clinical practice. The platform also incorporates automated segmentation of other computed tomography-derived anatomical structures, such as teeth, maxillary complex, mandible, mandibular canals, dental implants, and pharynx [22, 26–28], which could further optimize digital workflows. The underlying AI techniques offer the potential to enhance various dental specialties with precision and efficiency. They could be adapted for tasks in dental practices, enabling accurate analysis and treatment planning, and improving outcomes across orthodontics, prosthodontics, implantology, complex restorative dentistry, and forensic dentistry [29]. Beyond the orthodontic and prosthodontic cases presented, our platform’s segmentation capabilities can enhance pre-surgical planning for implants and post-operative assessment of peri-implant bone levels [28]. In the future, implant planning and placement could be improved by accurately mapping the bone structure and nerve canals, reducing risks and increasing success rates. Our platform also has potential benefits for complex restorative dentistry by providing precise anatomical segmentation, aiding in detailed reconstructions and restorations. The successful use of AI in automated forensic CBCT segmentation suggests opportunities for transfer learning to improve accuracy and efficiency in dental identification [30]. Our platform’s ability to segment and analyze CBCT and intraoral scan images could significantly improve the accuracy and speed of dental identification. AI-driven comparisons of dental records facilitate the identification of individuals in forensic investigations, enhancing the reliability of forensic analyses [31].

Techniques such as saliency maps and gradient-based methods are crucial for visualizing the areas a model focuses on, enhancing the interpretability of AI systems [32]. This results in more precise and reliable dental care, ensuring that AI systems are better verified, trusted, and adopted in practice [33]. Hasany et al. proposed MiSuRe for generating saliency maps in image segmentation [34]. These maps can act as proxies for post-hoc model reliability, distinguishing correct from incorrect predictions. In image segmentation, the overlap between the ground truth and model output is subjective and varies by application. Saliency maps help determine which predictions to accept or reject based on visualized focus areas, potentially automating this process [34]. Future studies are expected to incorporate these techniques to provide valuable insights for dentists, aiding in model refinement and validating AI recommendations.

As this study describes training of the model based on data derived from a single intraoral scanner, further strengthening of the algorithm is planned by introducing scans from various institutions and scanner brands to increase generalizability. AI models require continuous supervision and an ongoing learning process to maintain their effectiveness. To enhance their generalizability, future work should focus on training the current model to recognize a wider range of intra-oral scanner types, diverse tooth morphologies, and various dental anomalies. Additionally, the model should learn to identify missing and restored teeth, different dental treatments, and cases of dental crowding. By expanding the model’s knowledge base, we can eventually apply this AI across all dental specialties and treatment concepts, ensuring its comprehensive applicability in the field of dentistry.

## Conclusions

The proposed 3D U-Net pipeline outperformed state-of-the-art methods for automated tooth segmentation on IOS images, delivering accurate, efficient, and consistent results. Its clinical applicability is enhanced by the use of an online cloud-based platform for automated segmentation and interactive refinement.

## Data Availability

The datasets used and/or analysed during the current study are available from the corresponding author on reasonable request.

## Abbreviations

2D: Two-dimensional
3D: Three-dimensional
A-AI: Automated artificial intelligence
AI: Artificial intelligence
CNN: Convolutional neural network
CPU: Central processing unit
CUDA: Compute unified device architecture
DSC: Dice similarity coefficient
FN: False negatives
GPU: Graphics processing unit
HD: Hausdorff Distance
ICC: Intra-class correlation coefficient
IOS: Intraoral scanned
IoU: Intersection over Union
R-AI: Refined artificial intelligence
ROI: Region of interest
SA: Semi-automatic
SD: Standard deviation
STL: Standard tessellation language
TN: True negatives
TP: True positives
